# Insecticidal Efficacy of *Metarhizium anisopliae* Derived Chemical Constituents against Disease-Vector Mosquitoes

**DOI:** 10.3390/jof8030300

**Published:** 2022-03-15

**Authors:** Perumal Vivekanandhan, Kannan Swathy, Amarchand Chordia Murugan, Patcharin Krutmuang

**Affiliations:** 1 Society for Research and Initiatives for Sustainable Technologies and Institutions, Grambharti, Amarapur, Gujarat-382735, India; swathykannan.23@gmail.com (K.S.); amarchand.chordia@gmail.com (A.C.M.); 2 Department of Entomology and Plant Pathology, Faculty of Agriculture, Chiang Mai University, Chiang Mai 50200, Thailand; 3 Innovative Agriculture Research Center, Faculty of Agriculture, Chiang Mai University, Chiang Mai 50200, Thailand; 4 Research Center of Microbial Diversity and Sustainable Utilization, Faculty of Science, Chiang Mai University, Chiang Mai 50200, Thailand

**Keywords:** *Metarhizium anisopliae*, *Artemia nauplii*, *Eudrilus eugeniae*, mosquitoes, target specific, green pesticides

## Abstract

Insecticides can cause significant harm to both terrestrial and aquatic environments. The new insecticides derived from microbial sources are a good option with no environmental consequences. *Metarhizium anisopliae (mycelia) ethyl acetate extracts were tested on larvae, pupae, and adult of Anopheles stephensi* (Liston, 1901), *Aedes aegypti* (Meigen, 1818), *and Culex quinquefasciatus* (Say, 1823), *as well as non-target species Eudrilus eugeniae* (Kinberg, 1867) *and Artemia nauplii* (Linnaeus, 1758) at *24 h post treatment under laboratory condition*. In bioassays, *Metarhizium anisopliae* extracts had remarkable toxicity on all mosquito species with LC_50_ values, 29.631 in *Ae. aegypti*, 32.578 in *An. stephensi* and 48.003 in Cx. *quinquefasciatus* disease-causing mosquitoes, in *A. nauplii* shows (5.33–18.33 %) mortality were produced by the *M. anisopliae* derived crude extract. The LC_50_ and LC_90_ values were, 620.481; 6893.990 μg/mL. No behavioral changes were observed. A low lethal effect was observed in *E. eugeniae* treated with the fungi metabolites shows a 14.0 % mortality. The earthworm *E. eugeniae* mid-gut histology revealed that *M. anisopliae* extracts had no more harmful effects on the epidermis, circular muscle, setae, mitochondrion, and intestinal lumen tissues than chemical pesticides. By Liquid chromatography mass spectrometry (LC-MS) analysis, camphor (25.4 %), caprolactam (20.68 %), and monobutyl phthalate (19.0 %) were identified as significant components of *M. anisopliae* metabolites. Fourier transform infrared (FT-IR) spectral investigations revealed the presence of carboxylic acid, amides, and phenol groups, all of which could be involved in mosquito toxicity. The *M. anisopliae* derived chemical constituents are effective on targeted pests, pollution-free, target-specific, and are an alternative chemical insecticide.

## 1. Introduction

Mosquitoes are a major health problem because they transmit diseases such as malaria, dengue fever, yellow fever, and the Zika virus, which impact 700 million people each year and kill over one million people [1]. Adult mosquito control has been the primary approach for avoiding disease transmission, and it usually requires the use of synthetic pesticides and repellents, primarily organophosphates and pyrethroids [2]. Mosquitoes have evolved resistance to organophosphate and synthetic pyrethroids [3,4,5,6]. The chemical pesticides have been accumulated in our green ecosystem soil and waterbodies as well as food chains [5]. For insect pest management, entomopathogenic bacteria, fungi, and nematodes are considered effective microbial control methods for insect pests [7,8,9].

Entomopathogenic fungi produce secondary metabolites that could be used as a source for biopesticide development [10,11,12,13,14]. The entomopathogenic fungi *Metarhizium anisopliae* secondary metabolites, in particular, are known to be effective biopesticides for the control of *Aedes aegypti (Meigen, 1818)* mosquitoes [7,15], and other fungi such as *Tolypocladium* [13], *Beauveria* [9], *Fusarium* [10,11], and Lagenidium *giganteum* [14] have also been bioprospected as insect pests. Several biopesticides have been hampered by the fact that they are slow acting, taking anywhere from a few days to a week to exhibit action, which has hampered their commercialization [16]. Furthermore, their effects on non-target organisms are understudied [7].

The brine shrimp, *Artemia* sp. (Anostraca: Artemiidae), is a branchiopod crustacean that can tolerate salinities of up to 250 gL^−1^/L [17]. *Artemia* are commonly employed for the evaluation of marine contamination by synthetic chemicals because of their high sensitivity to chemicals or other toxicants [17], and *Artemia nauplii* (Linnaeus, 1758), an important component of the aquatic ecosystem, are regarded as indicators for environmental toxicity [17,18]. Earthworms, therefore, are considered to be bio-indicators of terrestrial ecosystems and are frequently used as biomarkers for assessing the environmental toxicity of chemical contaminants [19,20]. In the present study, we investigated the toxicity of secondary metabolites extracted from *Metarhizium anisopliae* (Metschn, 1879) strains and their toxicity effect was evaluated against disease-vector mosquitoes *Aedes Aegypti* (Meigen, 1818), *Anopheles Stephensi* (Liston, 1901), and *Culex quinquefasciatus* *(Say, 1823)*, as well as their toxicity against non-target organisms, such as earthworm *Eudrilus eugeniae* (Kinberg, 1867) *and brine shrimp Artemia nauplii* (Linnaeus, 1758).

## 2. Materials and Methods

### 2.1. Fungal Cultures

*M. anisopliae*, was isolated and collected from a soil sample from the Eastern Ghats of Tamil Nadu, India (Latitudes 11°30′ and 22° N, and longitudes 76°50′ and 86°30′ E). Morphological and 18s rDNA sequencing was used to identify fungi cultures. The gene sequences were submitted to the National Center for Biotechnology Information (NCBI, Data Base Accession No is: MH165400.1).

### 2.2. Mass Culturing of M. anisopliae 

*Metarhizium anisopliae* was cultured on Potato Dextrose Broth (PDB), as a medium for fungal growth. Sixteen 500 mL conical flasks, each containing 250 mL of PDB (dextrose 8 g, peptone 2 g and distilled water 250 mL), were autoclaved at 15 psi for 25 min. Chloramphenicol antibiotics (150 mg/mL) (Sigma-Aldrich Chemicals Private Limited, Bangalore) was added to the culture medium to prevent bacterial contamination. The cultures were allowed to grow for 20 days, and spore concentration was counted using a hemocytometer. A concentration of 1 × 10^7^ spores/mL of *M. anisopliae* conidia were transferred to the culturing medium using an inoculation needle. The culture medium was maintained at the optimized culture conditions (pH 7.0, temperature 28 ± 5 °C) for 30 days. 

### 2.3. Extraction of Secondary Metabolites

Fungus mycelial biomass was washed with distilled water after 20 days to eliminate culture medium components. *Metarhizium anisopliae* biomass was cold extracted with ethyl acetate to extract the biologically active chemical constituents under laboratory conditions. The ethyl acetate solvent was fully pooled with fungal biomass and left for 25 days. Then, the organic phase (light-yellow color) was separated after 25 days using a separating funnel, and the solvent was evaporated using a rotary evaporator at 45 °C.

### 2.4. Larval Collection and Maintenance

The Institute of Vector Control and Zoonoses at Hosur, Tamil Nadu, India. The mosquito egg masses per species were separately placed in a plastic tray (22 cm × 27 cm × 12 cm) (wonder, India) in dechlorinated tap water. The containers were transferred to room temperature with 28 ± 2 °C, 70–80% RH relative humidity and 12:12 (L:D) photoperiod and kept in it for 10–15 days. Each stage (larvae, pupae, and adult) of the mosquitos were taken for bioassay. During this process, mosquito larvae were fed with 0.5 g Tetra Bit (Pellet Fish Food) in each container, and adults were given a 10% sugar solution as a feeding source.

### 2.5. Non-Target Organisms

The *Eudrilus eugeniae* stock were maintained under laboratory condition at a room temperature of 27 ± 2 °C. *Artemia nauplii* larvae were kept in 1000 mL of saltwater with a salinity of 30 ppt in a culture medium with a pH range of (7–8). An aspirator was used to provide oxygen.

### 2.6. Mosquitocidal Bioassays

The fungal metabolites larvicidal and pupicidal efficacy was assessed using the World Health Organization protocol [21]. Stock solutions of fungal extract were dissolved in Dimethylsulfoxide (DMSO) (Sigma-Aldrich, India) at a concentration of 10% *w*/*v* (10 µg of extracts in 100 mL of DMSO) and diluted to five different concentrations: 10, 15, 30, 50, and 75 µg/mL. Twenty-five 4th instar larvae and pupae were each transferred to 249 mL of tap water with 1 mL of different concentrations of fungal extract and replicated three times. Dead insects were counted 24 h. As a negative control, DMSO at a concentration of 10% *w/v* was used. 

The adulticidal activity was evaluated following methods described by the Centers for Disease Control and Prevention [22]. Twenty-five newly emerged adults of *A. aegypti*, *A. stephensi* and *C. quinquefasciatus* were exposed to different concentrations (25, 50, 100, 150 and 200 μg/mL) of *M. anisopliae* secondary metabolites. Metabolites solutions were dispensed to the screw cap bottle of 80 mL, and for solvent evaporation, it was air dried over-night. In the control treatment, adult mosquitoes were exposed to DMSO (0.1%). Mortality was recorded post 24 h of treatment. A cotton ball soaked with a 10% glucose solution was used as a food source for mosquitoes. Three replicates for each concentration were performed (n = 450). 

### 2.7. Non-Target Bioassays

The effects of fungal metabolites on earthworms *E. eugeniae* was tested in an artificial soil composed of 15% sphagnum peat, 25% kaolinite clay, and 77 % fine sand. To keep the pH at 5.9, a few drops of CaCO_3_ were added. The water content was reduced to 30% of the dry weight. Fungi metabolites from *M. anisopliae* were put into the artificial soil at concentrations of 50 g/mL and 75 µg/mL. The 15 *E. eugeniae* larvae were then moved to a plastic container (375 mm × 300 mm × 75 mm) containing 1 kg of sterile artificial soil, which was then sealed with a plastic lid to keep the worms from escaping. Dead worms were counted 24–h after exposure. Monocrotophos was used as a positive control, while the negative control was free of fungal metabolites. Each treatment was replicated three times.

On brine shrimp *A. nauplii*, the toxicity of fungal secondary metabolites was determined as follows: Mature *A. nauplii* were collected with a hand pipette and utilized in toxicity tests on *A. nauplii* with different concentrations of *M. anisopliae* secondary metabolites (10, 15, 30, 50, and 75 µg/mL). As a negative control, the DMSO solution was employed. After 24 h of treatment, the *A. nauplii* dead mortality was calculated. Each concentration was tested three times, with each replicate containing 25 mature *A. nauplii*.

### 2.8. Fourier Transformed Infrared Spectroscopy Analysis

FT-IR analysis was conducted for the identification of the functional groups presents in the crude fungal metabolites. Two mg of fungi metabolites were properly mixed in 75 mg KBr; KBr acts as a binding agent on cleaned micro mortar and pestle. The mixed component was made into KBr pellets formed at low pressure. The KBr pellets were taken for FT-IR analysis using a BRUKER FT-IR spectrometer. FT-IR spectra scanning range was from 500 to 4000 cm^−1^. 

### 2.9. Liquid Chromatography-Mass Spectrophotometer Analysis

The chemical components profiling of crude fungal extracts was completed through the use of a Bruker Daltonik Impact II ESI-Q-TOF system (Bremen, Germany), ready with a Bruker Daltonik Elute, Ultra High Performance Liquid Chromatography (UHPLC) system (Bremen, Germany), in each positive (M + H) and negative (M − H) electrospray ionisation modes. Chromatographic separation was carried out on a Bruker Daltonik (Bremen, Germany) C18 reversed segment column (2.1 mm, 1.8 m, 120) at 30 °C, with an autosampler temperature of 8 °C and a total run time of 20 min, using water/methanol (90:10%) as eluent with five mM ammonium formate and 0.1% formic acid. The crude extract was dissolved in 2.0 mL of DMSO, and the quantity was multiplied to 50 mL with acetonitrile prior to centrifugation at 4000 rpm for two min and injection. The composition of the samples became mounted with the aid of figuring out the m/z ratio when it comes to the retention length of the utilised standards.

### 2.10. Statistical Analysis

The mortality rate was corrected using Abbot formula [23]. The dead *A. nauplii*, mosquito larvae, pupae, and adults were counted separately 24 h after treatment, and LC_50_ and LC_90_ were estimated using probit analysis. The SPSS-16.00 programme [18] was used to conduct all of the analyses.

## 3. Results

### 3.1. M. anisopliae metabolites against Ae. aegypti, An. stephensi, Cx. quinquefasciatus Mosquitoes

**Figure 1:**
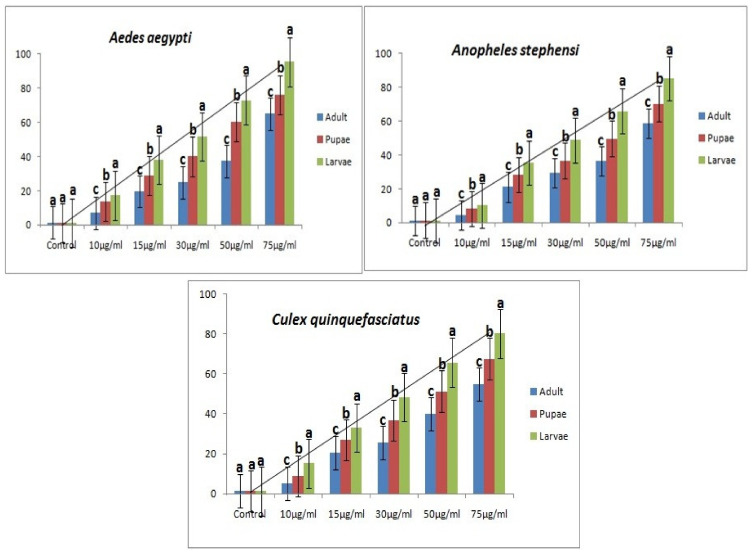
Larvicidal, pupicidal and adulticidal activities of *M. anisopliae* derived extract against larvae, pupae, and adult of *Ae. aegypti*, *An. stephensi* and *Cx. quinquefasciatus* vectors. Bars with the identical lower case letters do not differ significantly (*p* > 0.05).

*M. anisopliae* crude metabolites treatments, at the tested concentrations (10, 15, 30, 50 and 75 μg/mL), caused significant mortality against *Ae. aegypti* larvae (ranging from 17.33 to 95.33%), pupae (ranging from 13.66 to 76.00%), and adults (ranging from 7.00 to 65.00%) (Figure 1; Table 1). The probit model indicated that, *Ae. aegypti* larvae are more susceptible to the *M. anisopliae* crude metabolites than the pupae and adults with an LC_50_-29.631, 45.530, and 62.589 µg/mL for larvae, pupae, and adults, respectively (Table 1).

Mortality of *An. stephensi* larvae varied from 10.33 to 85.33%, for pupae, 8.33 to 70.33%, and adult (from 4.33 to 58.66%) (Figure 1; Table 1). As for *An. stephensi* the susceptibility of the larvae to the *M. anisopliae* crude metabolites was higher than the pupae and adults (LC_50_ = 32.578, 52.491, and 70.235µg/mL for larvae, pupae, and adults, respectively) (Table 1). Similarly, larvae mortality of *Cx. quinquefasciatus* varied from 8.66 to 80.33%; pupal from 6.00 to 61.00%, for adult 21.00 to 54.66% (Figure 1; Table 1). The toxicity of the *M. anisopliae* crude metabolites was higher for the *Cx. quinquefasciatus* larvae, (LC_50_ = 48.003µg/mL) than it was for the pupae (LC_50_ = 69.017µg/mL) or for the adults (LC_50_, 73.937µg/mL) (Table 1).

### 3.2. Non-Target Organisms

**Figure 2:**
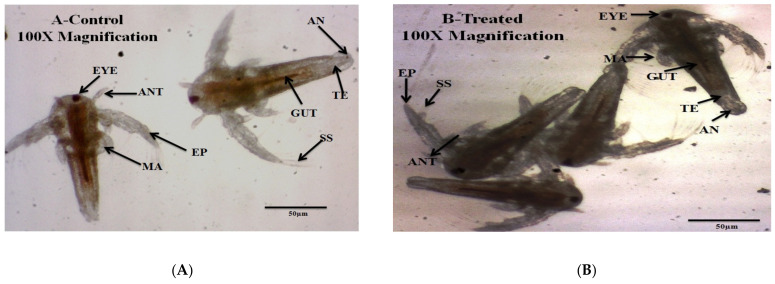
Morphological changes of *A. nauplii* exposed of *M. anisopliae* secondary metabolites at post 24 h of treatment. (**A**). Control (not treated fungal extract), (**B**). *M. anisopliae* secondary metabolites treated *A. nauplii* have no morphological changes were observed. (AN-1: Antennae 1, AN-2: Antennae 2, EYE: eye, EP: exopod, MA: mandible, GUT: gut, TE: telson, AN: anus, SS: swimming setae, ANT: antenna).

Entomopathogenic fungi *M. anisopliae* constituents showed a minimal effect on non-targeted *A. nauplii*. This study clearly shows (5.33–18.33 %) mortality were produced by the *M. anisopliae* derived crude extract (Table 2; Figure 2). The LC_50_ and LC_90_ values were 620.481; 6893.990 μg/mL (Table 2). No behavioral changes were observed during the treatment with fungal extracts.

**Figure 3:**
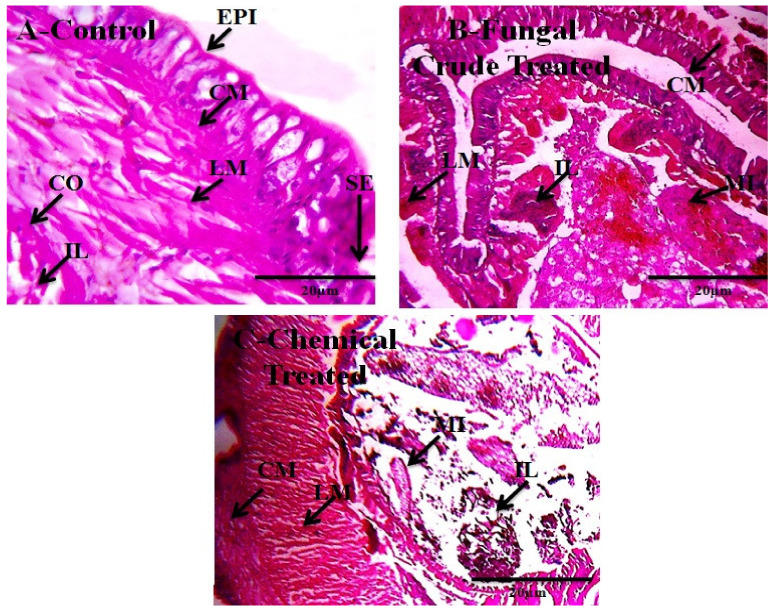
The *M. anisopliae* secondary metabolites (200 µg/mL) were exposed *E. eugeniae* and after 30 days of treatment, the earthworm gut tissues were sectioned for histopathological evaluation and magnified at 40× under a light microscope. (**A**) is control (without fungal crude extract treatment); (**B**) is fungal secondary metabolites treated; and (**C**) is Monocrotophos 200 ppm/kg treated. In the control and entomopathogenic fungi crude extract treatments, no changes were observed, but chemical pesticide treatment of several gut tissues morphology and shapes changed in the lumen tissues was entirely spoiled compared with control (EPI-epidermis, SE-setae, IL-intestinal lumen, LM-longitudinal muscle, CO-coelom, CM-circular muscle, MI-mitochondrion).

A low lethal effect was observed in *E. eugeniae* treated with the fungi metabolites; 14.0% mortality were observed in those treated with *M. anisopliae* secondary metabolites at 30 days after treatments. The highest earthworm mortality was observed in Monocrotophos pesticide treatment that shows 87.33 % mortality. Furthermore, the chemical treatment epidermis, intestinal and body wall thickness was reduced by the chemical (Figure 3; Table 3 and Table 4).

### 3.3. LC-MS and FT-IR Analysis

**Figure 4:**
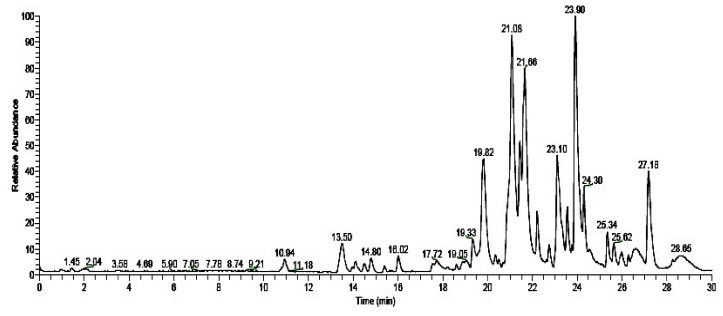
Chemical constituents were identified from *M. anisopliae* secondary metabolites using LC-MS analysis.

LC-MS analysis results of *M. anisopliae* extract showed the presence of two major chemical constituents, and retensition time namely Camphor (21.08), Caprolactam (21.66) and Monobutyl phthalate (23.90) (Figure 4; Table 5).

**Figure 5:**
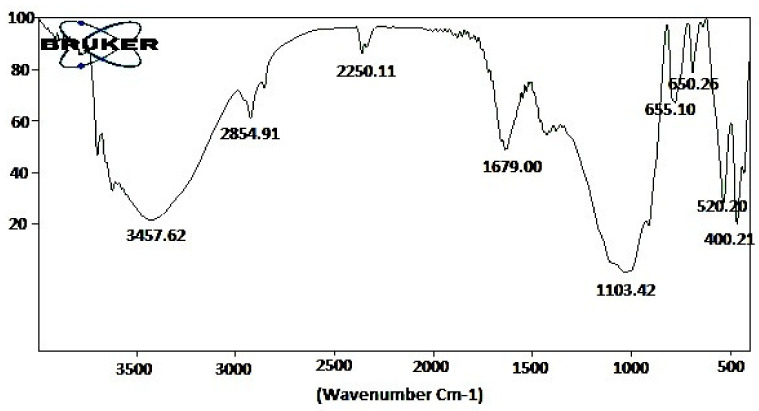
The major functional group was identified from *M. anisopliae* secondary metabolites using FT-IR analysis.

FT-IR showed the presence of functional groups such as, O–H stretching (3457.62 cm^−1^), O–H stretching (2854.91 cm^−1^) and the medium peak C=O stretching (1679.00 cm^−1^) (Figure 5; Table 6).

## 4. Discussion

Recently, there has been a great interest in the use of biologically derived pesticides as an alternative to synthetic chemicals [9,10]. Entomopathogenic fungi-derived toxins have several advantages over synthetic pesticides in that they kill mosquitos at different stages in both laboratory and environmental conditions, have lower toxic effects on non-target organisms, and remain stable for several months in extreme cold and hot conditions [9,10,14]. In this study, we evaluated the toxic effects of secondary metabolites isolated from *M. anisopliae* strains against larvae, pupae, and adults of the disease-vector mosquitoes *Ae. aegypti*, *An.* *stephensi* and *Cx. quinquefasciatus*, and we assessed their target specificity and environmental safety by testing the extracts against the aquatic and terrestrial non-target species *A. naupli* L. and *E. eugeniae*.

Fungal secondary metabolites showed clear toxicity against all the tested instars of the mosquitoes and much lower toxicity against the non-target organisms. In the present study, *M. anisopliae* crude metabolites showed high toxicity towards the larvae, pupae, and adults of *A. aegypti*, *A. stephensi* and *C. quinquefasciatus* mosquitoes at 24 h post treatment under laboratory conditions (Figure 1; Table 1). In line with our results, previous studies on entomopathogenic fungal derived pesticides from several species of *Metarhizium*, *Fusarium*, *Aspergillus*, *Trichoderma* and *Lecanicillium* showed that they are effective against medical and agricultural insect pests [24]. Soni and Prakash [25] reported that *Chrysosporium* *keratinophilum* derived secondary metabolites have strong larvicidal activity against *C. quinquefasciatus* and *A. stephensi* mosquito larvae, while [26] reported that different fungal metabolites cause strong larvicidal activity against larvae of *A. stephensi* and *C. quinquefasciatus*. Similarly, *Metarhiziumanisopliae*, *Aspergillus flavus*, *Fusarium oxysporum*, *Verticillium lecanii*, *Paecilomyces fumosoroseus*, *Beauveria bassiana*, and *Fusarium moniliforme* and their toxins have been shown to produce remarkable mosquitocidal potential on larvae, pupae, and adult mosquitoes [9,10,11,27]. *C. tropicum*, *C. clavisporus* and *F. oxysporum* culture filtrates showed strong larvicidal activity against *A. stephensi*, *A. aegypti* and *C. quinquefasciatus* [10,11,22,28], and secondary metabolites of *A. fumigatus* showed strong larvicidal activity against larvae of *A. aegypti* [29].

On the contrary, in our study, we observed low toxicity of the fungal metabolites against non-target species such as *A. nauplii* and *E. eugeniae* (Table 2, Table 3 and Table 4; Figure 2 and Figure 3). Similarly, [30] reported few swimming speed alterations in *Artemia* adults after their treatment by different toxins. A similar study about the effects of the fungi secondary metabolites from *Penicillium daleae* on *Artemia,* observed morphological changes in eye shape, eye color, and eye fading [31]. These results suggest that secondary metabolites from different fungi may produce lower levels of toxicity to non-target organisms. For this reason, the assessment of the lower effects of fungal secondary metabolites in aquatic and terrestrial ecosystems on non-target species is of prime importance. The chemical composition of the secondary metabolites extracted from the *M. anisopliae* entomopathogenic fungi analysed in this study is in accord with previous research by [9,10] and by [32], who observed similar kinds of chemical constituents (Figure 4; Table 5). Previously, [9,10] reported that *B. bassiana* and *F. oxysporum* derived crude metabolites had the same chemical constituents showing a strong larvicidal activity on *A. aegypti*, *A. stephensi* and *C. quinquefasciatus* larvae. In this study, FT-IR analyses showed the presence of phenols, biogenic amines, and carboxylic acids, which may be involved in the toxic effects on mosquitoes (Figure 5; Table 6). 

Similarly, previous studies showed that the metabolites of entomopathogenic fungi are constituted by components belonging to several chemical classes (phenols, alcohols, carboxylic acids, misc, aromatics, phosphoramide, and disulfides), which may be involved in the mosquitocidal effects [9,10,11,26,33,34,35,36]. The strong mosquitocidal activity and the low toxic effect on non-target organisms exhibited by *M. anisopliae* indicate that, besides entomopathogenic fungal conidia, their metabolites may also have a significant role in efficient microbial-derived mosquito control tools that can be used in mosquito control programmes as effective, cheaper, biodegradable, target-specific alternatives to chemical insecticides. Further research into the single crude metabolite chemical constituents under laboratory and semi-field conditions may result in the development of effective *M. anisopliae* derived bio-pesticides.

## 5. Conclusions

The strong mosquitocidal activity and the low toxic effect on non-target organisms exhibited by *M. anisopliae* indicate that, besides entomopathogenic fungal conidia, their metabolites may also have a significant role in efficient microbial-derived mosquito control tools that can be used in mosquito control programmes as effective, cheaper, biodegradable, target-specific alternatives to chemical insecticides. Further research into the single crude metabolite chemical constituents under laboratory and semi-field conditions may result in the development of effective *M. anisopliae* derived biopesticides.

## Figures and Tables

**Table 1:** Mosquitocidal activities of *M. anisopliae* ethyl acetate crude extract against larvae, pupae, and adults of three mosquito species at 24 h after treatments.

Mosquito	Stage	N = Insect Number	LC_50_ (LCL-UCL)	LC_90_ (LCL-UCL)	χ^2^ (df = 12)
*Ae. aegypti*	Larvae	450	29.631 (25.440–36.833)	80.560 (74.910–87.001)	5.673
	Pupae	450	45.530 (39.920–51.532)	103.430 (98.571–109.642)	4.041
	Adult	450	62.589 (57.439–67.991)	123.775 (115.679–129.002)	6.090
*An. stephensi*	Larvae	450	32.578 (27.871–35.900)	88.003 (82.717–93.966)	5.214
	Pupae	450	52.491 (46.913–56.331)	98.110 (95.332–105.88)	1.287
	Adult	450	70.235 (66.057–75.339)	150.921 (141.883–157.991)	3.002
*Cx. quinquefasciatus*	Larvae	450	48.003 (41.771–53.994)	96.883 (93.880–103.439)	6.454
	Pupae	450	69.017 (64.771–74.000)	158.881 (151.875–164.640)	0.989
	Adult	450	73.937 (66.383–78.382)	180.440 (176.003–189.337)	7.046

na is total number of larvae, pupae and adult used per each species, 25 per replicate, three replicates were carried out, five concentrations were tested; LC_50_ = lethal concentration killing 50% of exposed organisms; LC_90_ = lethal concentration killing 90% of exposed organisms; LCL = 95% lower confidence limits; UCL = 95% upper confidence limits; χ^2^ = chi square; df = degrees of freedom; SD = Standard deviation.

**Table 2:** Toxicity of *M. anisopliae* secondary metabolites on *A. nauplii* at 24 h after treatments.

Mosquito (na = 450)	Concentration (µg/mL)	% Mortality ± SD	LC_50_ (LCL-UCL)	LC_90_ (LCL-UCL)	χ^2^ (df = 12)
*M. anisopliae*	Control	1.33 ± 0.5	620.481 (612.550–635.779)	6893.990 (6587.612–7432.900)	1.599
	10	5.33 ± 0.5			
	15	12.66 ± 1.0			
	30	15.0 ± 0.5			
	50	13.33 ± 1.0			
	75	18.33 ± 0.5			

na = total number of *A. nauplii* used per each species, 25 per replicate, three replicates were carried out, five concentrations were tested; LC_50_ = lethal concentration killing 50% of exposed organisms; LC_90_ = lethal concentration killing 90 % of exposed organisms; LCL = 95 % lower confidence limits; UCL = 95 % upper confidence limits; χ^2^ = chi square; df = degrees of freedom; SD = Standard deviation.

**Table 3:** Mortality of *E. eugeniae* after the treatment of *M. anisopliae* crude extract and Monocrotophos at post 24 h treatments. The identical lower case letters do not differ significantly (*p* > 0.05).

Treatment	Concentration (µg/mL)	% Mortality ± SD
*M. anisopliae*	Control	1.33 ± 0.5 ^a^
	50	4.66 ± 1.0 ^b^
	75	14.00 ± 1.1 ^c^
Monocrotophos	Control	1.33 ± 0.5 ^a^
	50	50.00 ± 0.5 ^b^
	75	87.33 ± 0.5 ^c^

**Table 4:** Thickness of the epidermis, intestinal epithelium, and body wall of earthworms after the 30 days treatment of *M. anisopliae* crude extract. The identical lower case letters do not differ significantly (*p* > 0.05).

Treatments	*E. eugeniae*		
	Epidermis (µm) ± SD	Intestinal Epithelium (µm) ± SD	Body Wall (µm) ± SD
Control	37.13 ± 0.0 ^a^	71.14 ± 0.5 ^a^	280.12 ± 0.0 ^a^
*M. anisopliae*	36.51 ± 0.5 ^b^	70.55 ± 0.5 ^b^	279.10 ± 0.0 ^b^
Monocrotophos	23.32 ± 0.5 ^c^	55.15 ± 1.1 ^c^	210.12 ± 0.5 ^c^

**Table 5:** The *M. anisopliae* ethyl acetate crude extract chemical constituents were identified using LC-MS analysis.

S. No	Retention Time	Molecular Formula	Molecular Weight	Compound Name	Compound Structure
1	19.82	C_37_H_67_NO_13_	733.46124	(-)-Erythromycin	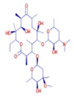
2	21.08	C_10_H_16_O	152.12012	(-)-Camphor	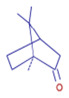
3	21.66	C_6_H_11_NO	113.08406	Caprolactam	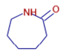
4	23.10	C_16_H_30_O_4_	286.21441	2,2,4-Trimethyl-1,3-pentadienol diisobutyrate	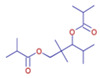
5	23.90	C_12_H_14_O_4_	222.08921	Monobutyl phthalate	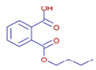
6	24.30	C_20_H_38_O_2_	310.28718	Ethyl oleate	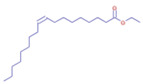
7	27.18	C_16_H_22_O_4_	278.15181	Dibutyl phthalate	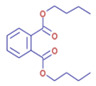

**Table 6:** The major functional group was identified from *M. anisopliae* ethyl acetate crude extract using FT-IR analysis.

S. No	Observed Wavenumber (cm^−1^)	Functional Group	Bonding Pattern
1	3457.62	O–H stretch	Phenols
2	2854.91	O–H stretch	Carboxylic acids
3	2250.11	-C C- stretch	Alkynes
4	1679.00	C=O stretch	Aldehydes
5	1103.42	C-H wag	Alkyl halides
6	655.10	C-H bends	Aromatics
7	650.25	C-H bends	Aromatics
8	520.20	C-Br stretch	Alkyl halides
9	400.21	C-Br stretch	Alkyl halides

## Data Availability

The data that support the findings of this present study are available from the corresponding author upon reasonable request.
